# Chicken IFI6 inhibits avian reovirus replication and affects related innate immune signaling pathways

**DOI:** 10.3389/fmicb.2023.1237438

**Published:** 2023-11-16

**Authors:** Lijun Wan, Sheng Wang, Zhixun Xie, Hongyu Ren, Liji Xie, Sisi Luo, Meng Li, Zhiqin Xie, Qing Fan, Tingting Zeng, Yanfang Zhang, Minxiu Zhang, Jiaoling Huang, You Wei

**Affiliations:** ^1^Guangxi Key Laboratory of Veterinary Biotechnology, Guangxi Veterinary Research Institute, Nanning, Guangxi, China; ^2^Key Laboratory of China (Guangxi)-ASEAN Cross-Border Animal Disease Prevention and Control, Ministry of Agriculture and Rural Affairs of China, Nanning, Guangxi, China

**Keywords:** IFI6, structure, function, antiviral, ARV, innate immune signaling pathway

## Abstract

Interferon-alpha inducible protein 6 (IFI6) is an important interferon-stimulated gene. To date, research on IFI6 has mainly focused on human malignant tumors, virus-related diseases and autoimmune diseases. Previous studies have shown that IFI6 plays an important role in antiviral, antiapoptotic and tumor-promoting cellular functions, but few studies have focused on the structure or function of avian IFI6. Avian reovirus (ARV) is an important virus that can exert immunosuppressive effects on poultry. Preliminary studies have shown that IFI6 expression is upregulated in various tissues and organs of specific-pathogen-free chickens infected with ARV, suggesting that IFI6 plays an important role in ARV infection. To analyze the function of avian IFI6, particularly in ARV infection, the chicken IFI6 gene was cloned, a bioinformatics analysis was conducted, and the roles of IFI6 in ARV replication and the innate immune response were investigated after the overexpression or knockdown of IFI6 *in vitro*. The results indicated that the molecular weight of the chicken IFI6 protein was approximately 11 kDa and that its structure was similar to that of the human IFI27L1 protein. A phylogenetic tree analysis of the IFI6 amino acid sequence revealed that the evolution of mammals and birds was clearly divided into two branches. The evolutionary history and homology of chickens are similar to those of other birds. Avian IFI6 localized to the cytoplasm and was abundantly expressed in the chicken lung, intestine, pancreas, liver, spleen, glandular stomach, thymus, bursa of Fabricius and trachea. Further studies demonstrated that IFI6 overexpression in DF-1 cells inhibited ARV replication and that the inhibition of IFI6 expression promoted ARV replication. After ARV infection, IFI6 modulated the expression of various innate immunity-related factors. Notably, the expression patterns of MAVS and IFI6 were similar, and the expression patterns of IRF1 and IFN-β were opposite to those of IFI6. The results of this study further advance the research on avian IFI6 and provide a theoretical basis for further research on the role of IFI6 in avian virus infection and innate immunity.

## Introduction

Innate immunity is the body’s first line of immune defense against pathogen invasion. After infection of a host, a virus is sensed by pattern recognition receptors, and this sensing triggers a series of intracellular signaling pathways and induces the expression and secretion of a variety of cytokines, such as proinflammatory factors, chemokines, and interferons (IFNs) (Ivashkiv and Donlin, 2014). As important cytokines, IFNs mediate an antiviral response and play an extremely important role in innate immunity (William et al., 2014). IFNs themselves exert no antiviral effects; in contrast, IFNs induce antiviral activity by inducing the expression of a variety of interferon-stimulated genes (ISGs) (Borden et al., 2007). Therefore, in-depth research on the expression of ISGs and their mechanisms of action is expected to increase the understanding of host antiviral immune responses and suggest distinct drug targets in the treatment of specific infectious diseases (Ivashkiv and Donlin, 2014). Hundreds of ISGs have been identified thus far, and these play important roles in host antiviral infection responses, including immune system regulation. The gene encoding interferon-alpha inducible protein 6 (IFI6), which is also known as G1P3 and IFI6-16, is an important ISG. IFI6 expression can be upregulated by type I IFNs and belongs to the FAM14 family (Parker and Porter, 2004; Cheriyath et al., 2011). IFI6 is found only in eukaryotes. Studies have shown that human IFI6 is located on chromosome 1 and comprises 812 bp that encode 130 amino acids with a molecular weight of approximately 13 kDa. The protein structure of IFI6 is sequentially composed of signal peptides in the N-terminus, a hydrophilic region, a transmembrane region, a connecting region, a transmembrane region, and a hydrophilic region (Cheriyath et al., 2011). The structure and function of human IFI6 are clearly understood, and studies have shown that IFI6 plays an important role in a variety of human diseases. In recent years, several studies have shown that IFI6 is associated with a variety of malignant diseases; notably, IFI6 is highly expressed in various malignant tumors, virus-related diseases and autoimmune diseases (Tahara et al., 2005; Cheriyath et al., 2007). Further research has revealed that IFI6 plays an important role in many processes, such as antiviral, antiapoptotic and tumor-promoting activities. For example, IFI6 is highly expressed in gastric cancer cell lines and tissues, is enriched mainly in the inner mitochondrial membrane, colocalizes with cytochrome c in mitochondria, and can inhibit caspase-3 activity by inhibiting mitochondrial membrane depolarization and the release of cytochrome c, which results in the inhibition of apoptosis (Tahara et al., 2005). Other studies have found that the expression of IFI6 is significantly elevated in esophageal squamous cell carcinoma (ESCC) patients and ESCC cell lines cultured *in vitro*. Further in-depth studies have revealed that high expression of IFI6 in ESCC cells exerts an oncogenic effect and that the knockdown of IFI6 expression increases the accumulation of reactive oxygen species, which leads to inhibition of the proliferation of cancer cells and induction of apoptosis (Liu et al., 2020). In terms of its antiviral activity, IFI6 confers protection to uninfected cells by blocking yellow fever virus-, West Nile virus-, dengue virus- and Zika virus-induced endoplasmic reticulum membrane invagination; i.e., IFI6 interacts with Bip, which is a companion to endoplasmic reticulum heat shock protein 70 encoded by HSPA5, to prevent virus-induced endoplasmic reticulum membrane invagination and protect uninfected cells and thereby achieves antiviral effects (Richardson et al., 2018). Studies of influenza A virus, severe acute respiratory syndrome coronavirus 2, and Sendai virus found that IFI6 negatively regulates the innate immune response induced by these viruses by affecting RIG-1 activation (Villamayor et al., 2023). However, little is known about the role of avian IFI6 in avian diseases; therefore, the structure and function of avian IFI6 are worth exploring.

Avian reovirus (ARV), which is a double-stranded RNA virus that exists widely in nature and can infect chickens, ducks, geese, turkeys and other birds, has been detected in some wild birds (Olson, 1959; van der Heide, 2000; Zhang et al., 2019). ARV infection can cause viral arthritis/tenosynovitis, short stature syndrome, and malabsorption syndrome (Czekaj et al., 2018). In addition, ARV infection can lead to immunosuppression, causing cell damage in various immune-related organs, such as the bursa of Fabricius, thymus, and spleen of chickens (Sharma et al., 1994). ARV infection makes the host more susceptible to infection with other pathogens, resulting in increased mortality due to coinfection and posing serious threats to healthy poultry development and to the poultry industry (Roessler and Rosenberger, 1989). Innate immunity plays an important role in host defense against ARV infection, and considerable research has investigated ARV and the innate immunity of hosts. Studies have found that ARV infection activates the expression of many innate immune response-related factors in a host. ARV S1133 infection in specific-pathogen-free chickens can induce changes in the expression of various innate immunity-related factors, including IFN-α, IFN-β, IFN-γ, IL-6, IL-17, IL-18, MX, IFITM3, PKR, OAS, IFIT5, ISG12, VIPERIN, and IFI6, in chicken peripheral blood lymphocytes and joints (Xie et al., 2019; Wang et al., 2021, 2022). In addition, many studies have aimed to understand the intracellular molecular mechanisms underlying the effects of ARV infection. The host protein NME2 affects the replication of ARV in chicken embryo fibroblasts (CEFs) by binding to the structural ARV protein σA (Xie et al., 2021); ARV induces apoptosis by activating UPR-related signaling pathways through ATF6 (Zhang et al., 2021); and in CEFs, ARV induces IFN production through caspases (Lostale-Seijo et al., 2016).

In this study, we cloned the chicken IFI6 gene, performed bioinformatics analysis and subcellular localization assays of IFI6, determined the expression distribution of IFI6 in different chicken tissues, and investigated the effect of IFI6 on ARV replication and the expression of innate immunity signaling pathway-related factors after IFI6 overexpression and knockdown. The results of the study increase the understanding of the function of avian IFI6 and provide a theoretical basis for further studies on the pathogenic mechanism of the effects of ARV and for developing specific drugs to fight ARV infection.

## Materials and methods

### Ethics statement

All research and regulatory licenses were approved by the Animal Protection and Experiment Ethics Committee of Guangxi Veterinary Research Institute (approval number: 2019C0407).

### Chicken, cells, and virus

Specific-pathogen-free Bai Laihang chicken embryos were purchased from Merial Vital Laboratory Animal Technology Co., Ltd. (Beijing, China), and hatched using an automatic incubator. After hatching, the chicks were transferred to a specific-pathogen-free chicken incubator for rearing. Three 14-day-old specific-pathogen-free chickens that exhibited good growth were selected, and specimens from 15 tissues were collected: heart, liver, lung, bursa of Fabricius, thymus, spleen, intestine, glandular stomach, gizzard, muscle, trachea, brain, kidney, joint and pancreas. The specimens were snap frozen in liquid nitrogen and stored at −70°C. The virus strain ARV S1133 used in this study was purchased from the China Academy of Veterinary Drug Inspection (Beijing, China). DF-1 cells were cultured in Dulbecco’s modified Eagle’s medium: nutrient mixture F-12 (Gibco, USA) supplemented with 10% fetal bovine serum (Gibco), 100 μg/mL streptomycin sulfate and 100 U/mL penicillin sodium (Beyotime Biotechnology, China).

### Cloning and bioinformatics analysis of the IFI6 gene

The gene and amino acid sequences of IFI6 were downloaded from the National Center for Biotechnology Information (NCBI) database. Sequence alignment was performed, and primers (IFI6-U and IFI6-D) were designed (Table 1). The avian IFI6 gene was cloned using cDNA obtained by reverse transcription of mRNA extracted from DF-1 cells and used as the template. DNAStar, MEGA software and the online tools GOR4^1^ and SWISS-MODEL^2^ were used to perform the sequence alignment, phylogenetic tree construction, homology analysis, and secondary and tertiary structure prediction analysis based on the IFI6 gene sequence.

**TABLE 1:** Primers used in this study.

Primer name	Sequence (5′-3′)	Amplified sequence length
IFI6-U	GCGTCGACCATGTCTGA CCAGAACGTCCAC	324 bp
IFI6-D	GCGCGGCCGCTCAGCGCCT TCCTCCTTTGCCA	

### Plasmid construction and transfection

IFI 6-U and IFI6-D primers were designed using the pEF1α-Myc and IFI6 gene sequences (Table 1). The IFI6 gene was cloned and inserted into a pEF1α-Myc plasmid, and its accurate insertion was confirmed by RayBiotech (Guangzhou, China). Then, 2 μg of the correctly sequenced plasmid was transfected into 1 × 10^6^ DF-1 cells using a Lipofectamine™ 3000 Transfection Kit (Thermo Scientific, USA), and protein from DF-1 cells transfected with the pEF1α-Myc plasmid was used as the blank control. The overexpression of IFI6 was verified by Western blotting 48 h after transfection.

### RNA interference

Using the chicken IFI6 gene sequence, three specific short interfering RNAs (siRNAs) (si54, si156, and si274) and siRNAs directed to an unrelated molecule, siNC, were designed and synthesized by Suzhou GenePharma Co., Ltd. The siRNA sequences are shown in Table 2. Lipofectamine™ RNAiMAX Reagent (Thermo Scientific) was used for the transfection of 30 pmol of si54, si156, and si274 into DF-1 cells to inhibit IFI6 expression. Using siNC-transfected DF-1 cells as the control cells, we performed real-time fluorescence quantitative PCR to identify the siRNA that exerted the greatest inhibitory effect 24 h after transfection.

**TABLE 2:** Short interfering RNA (siRNA) sequences targeting the IFI6 gene.

siRNA	Sequence (5′-3′)	Sequence (3′-5′)
si54	GCAAGAGGUUCUCU UGCUUTT	AAGCAAGAGAACCUCU UGCTT
si156	GCCAAAGGCUCAAC ACACUTT	AGUGUGUUGAGCCUU UGGCTT
si274	GCACAACUUCCACUA UCCATT	UGGAUAGUGGAAGUU GUGCTT
siNC	UUCUCCGAACGUGUCA CGUTT	ACGUGACACGUUCGGA GAATT

### RNA extraction and real-time fluorescence quantitative PCR

Total RNA was extracted from cells and chicken tissue specimens using a Thermo Scientific GeneJET RNA Purification Kit (Thermo Scientific), and cDNA was synthesized by reverse transcription. The relative expression levels of IFI6, GAPDH, MAVS, IRF1, IRF7, STING, TBK1, NF-κB, MDA5, LGP2, IFN-α, IFN-β, and IFN-γ were quantified by real-time fluorescence quantitative PCR with specific primers as previously reported (He et al., 2016). Real-time fluorescent quantitative PCRs were performed in 96-well plates using PowerUp SYBR Green Master Mix (Thermo Scientific). The reaction conditions were as follows: 94°C for 2 min and 40 cycles of 94°C for 15 s, and 60°C for 30 s. Using the GAPDH housekeeping gene as the reference, the expression levels of target genes were normalized, and the relative expression levels of the genes were calculated using the 2^–ΔΔCT^ method.

### Protein extraction and western blotting

Forty-eight hours after transfection, the culture medium was discarded, and the cells were washed three times with 1 × PBS and then lysed in lysis buffer (Beyotime Biotechnology) on ice for 30 min. After lysis, the cell supernatant was obtained by centrifugation at 4°C and processed for use in SDS-PAGE. After electrophoresis, the protein was transferred to a polyvinylidene fluoride membrane, and the membrane was incubated with Western blot blocking solution for 6 h. The membranes were incubated with diluted mouse anti-Myc monoclonal antibody (Abcam, UK) and β-actin monoclonal antibody (Abcam) at 37°C for 2 h, and the membrane was then washed 3 times (10 min each time) with 1 × TBST buffer (Beyotime Biotechnology), incubated with HRP-labeled IgG (Beyotime Biotechnology) at 37°C for 1 h and washed 3 times with 1 × TBST buffer (Beyotime Biotechnology). Photographs were taken after color development using a 3,3′-diaminobenzidine (DAB) horseradish peroxidase color development kit (Beyotime Biotechnology).

### Confocal microscopy analysis of the subcellular localization of target proteins

The cells were cultured in a special dish designed for use in laser confocal microscopy. Forty-eight hours after transfection, the culture medium was discarded, and the cells were fixed with a 4% tissue cell fixative solution for 10 min, permeabilized with 0.1% Triton X-100 (Beyotime Biotechnology) for 15 min, and blocked with 5% bovine serum albumin (Beyotime Biotechnology) for 1 h. The cells were then incubated with diluted mouse anti-Myc monoclonal antibody (Abcam) at 37°C for 2 h, washed three times (5 min each) with 1 × TBST buffer (Beyotime Biotechnology) and incubated with Alexa Fluor 488-labeled goat anti-mouse IgG (H + L) cross-adsorbed secondary antibody (Invitrogen, USA) for 1 h at 37°C. After three washes with 1 × TBST buffer (Beyotime Biotechnology), DAPI (Beyotime Biotechnology) was added to stain the nuclei. The stain was discarded after 10 min, and the cells were washed 3 times (5 min each time) with 1 × PBS buffer (Beyotime Biotechnology). Subsequently, an appropriate amount of 1 × PBS buffer (Beyotime Biotechnology) was added to the cells, and the cells were then observed and photographed using a laser confocal microscope at 63 × magnification and excitation wavelengths of 405 nm and 488 nm.

### Statistical analysis

All data represent the results from at least three independent experiments. Statistical analyses were performed by *t*-test with GraphPad Prism 5.0 software. The results are presented as follows: * indicates *P* < 0.05, ** indicates *P* < 0.01, *** indicates *P* < 0.001, and **** indicates *P* < 0.0001.

## Results

### Cloning and bioinformatics analysis of IFI6

**FIGURE 1:**
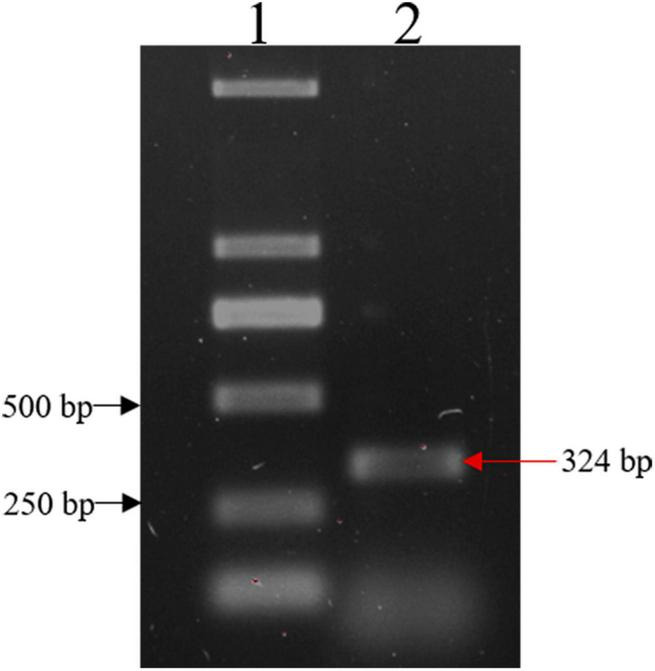
Cloning of the IFI6 gene. Lane 1: DL2000 DNA marker; lane 2: PCR amplification product. The red arrow indicates the amplified IFI6 fragment, which is 324 bp.

The IFI6 gene was amplified using cDNA reverse transcribed from mRNA extracted from DF-1 cells as a template and the primers IFI6-U and IFI6-R (Figure 1). The sequence was uploaded to the NCBI-Blast online website for alignment. The cloned sequence was confirmed to be the full-length sequence encoded by the IFI6 gene of *Gallus gallus* located on chicken chromosome 2, and its coding DNA sequence (CDS) consisted of 324 bases encoding a total of 107 amino acids.

Using DNAStar software, sequence homology analysis was performed with the full-length cloned sequence of the chicken IFI6 gene coding region and corresponding sequences from other species (Table 3). The similarity of the cloned sequence with the corresponding sequences of *Merops nubicus* and *Taeniopygia guttata* birds was generally higher than the similarity of the cloned sequence with the corresponding sequences of mammals.

**TABLE 3:** Sequence homology of the coding region of the IFI6 gene in different species.

Species	Accession number	Identity
*Merops nubicus*	XM_008944065.1	73.6%
*Taeniopygia guttata*	NM_001197179.2	87.4%
*Homo sapiens*	NM_022873.3	37.9%
*Bos taurus*	NM_001075588.1	40.0%
*Ornithorhynchus anatinus*	XM_029080218.2	34.8%
*Ovis aries*	XM_027965432.2	40.0%
*Canis lupus familiaris*	XM_535344.7	39.8%
*Sus scrofa*	XM_021095658.1	40.3%

**FIGURE 2:**
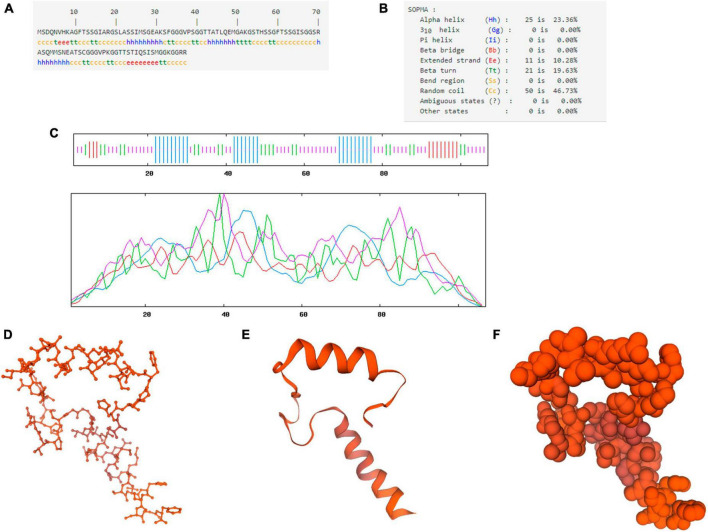
Structural prediction of the IFI6 protein. Primary structure showing the polypeptide chain encoded by the gene consisting of 107 amino acids **(A)**. Secondary structure **(B)**. Amino acid sequence analysis (blue represents alpha helices, purple represents random coils, red represents extended strands, and green represents beta turns) **(C)**. Prediction of the three-dimensional structural model of the IFI6 protein: backbone structure **(D)**, ribbon structure **(E)**, and spherical structure **(F)**. The global model quality estimation (GMQE) score for the predicted IFI6 protein structure and interferon alpha-inducible protein 27-like protein 1 (IFI27L1) structure was 0.30 and the coverage was 51%.

The primary structure analysis of the chicken IFI6 protein showed that the polypeptide chain encoded by the gene consisted of 107 amino acids and had a molecular weight of approximately 10.18 kDa (Figure 2A). GOR4 was used to predict the secondary structure of the IFI6 protein (Figure 2B): alpha helices, 23.36%; extended strands, 10.28%; beta turns, 19.63%; and random coils, 46.73%. The specific distribution is shown in Figure 2C, where blue represents alpha helices, purple represents random coils, red represents extended strands, and green represents beta turns.

SWISS-MODEL was used to construct a three-dimensional structural model of the amino acid sequence of the IFI6 protein (Figures 2D–F). The global model quality estimation (GMQE) score for the predicted IFI6 protein structure and interferon alpha-inducible protein 27-like protein 1 (IFI27L1) structure was 0.30 and the coverage rate was 51%.

National Center for Biotechnology Information, DNAStar and other software packages were used to analyze the homology between the amino acid sequence encoded by the chicken IFI6 gene and the corresponding amino acid sequences in other species. The homology of the amino acid sequences of chicken IFI6 with that of *Homo sapiens*, *Bos taurus*, *Ornithorhynchus anatinus*, *Ovis aries*, *Canis lupus familiaris*, *Sus scrofa*, *M. nubicus*, and *T. guttata* IFI6 was 29.6, 26.8, 21.8, 26.1, 26.8, 26.1, 67.6, and 83.1%, respectively.

**FIGURE 3:**
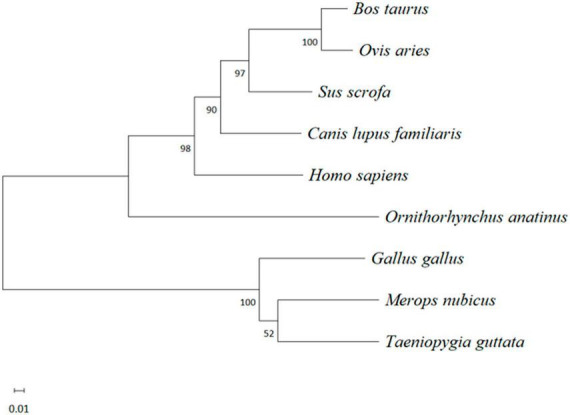
Evolutionary analysis of the amino acid sequence of IFI6 among different species. A phylogenetic tree was constructed using the neighbor-joining (NJ) method with MEGA software. The scale bars represent the branch lengths, and bootstrap confidence values are displayed at the nodes of the tree.

After multiple alignment of the amino acid sequences of the abovementioned species, a phylogenetic tree of the IFI6 amino acid sequence was constructed using MEGA software with the neighbor-joining (NJ) method and 1000 bootstrap replicates. The results are shown in Figure 3. Mammals and birds were clearly clustered in their respective categories, and *O. anatinus* was positioned between the two branches but closer to the mammalian branch.

### Subcellular localization

**FIGURE 4:**
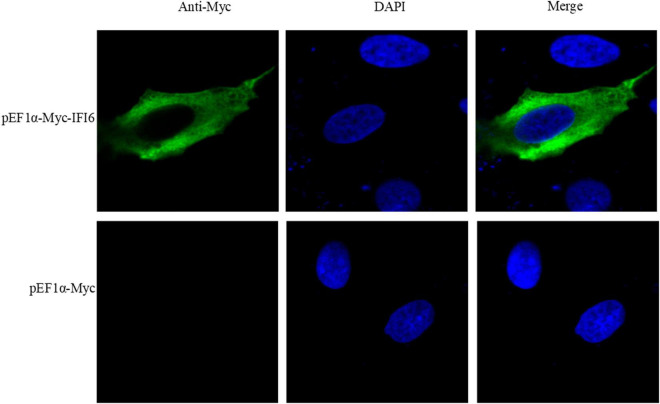
Subcellular localization of the IFI6 protein (63 × magnification). DF-1 cells were transfected with pEF1α-Myc-IFI6 or pEF1α-Myc. Forty-eight hours after transfection, the cells were incubated with a mouse anti-Myc monoclonal antibody for 2 h and then incubated with an Alexa Fluor 488 (green)-labeled goat anti-mouse IgG (H + L) cross-adsorbed secondary antibody for 1 h. Nuclei were stained with DAPI (blue), and the fluorescence intensity was detected by confocal microscopy and used to assess the location of expressed IFI6.

Alexa Fluor 488 (green)-labeled goat anti-mouse IgG (H + L) cross-adsorbed secondary antibody (Invitrogen) was used to label the IFI6 protein. Nuclei were stained with DAPI (blue) (Beyotime Biotechnology). The subcellular localization of the IFI6 protein was observed by laser confocal microscopy, and the results are shown in Figure 4. DF-1 cells transfected with a pEF1α-Myc-IFI6 plasmid emitted green fluorescence throughout the cytoplasm, indicating that the IFI6 protein localized to the cytoplasm. After receiving the same treatment, DF-1 cells transfected with an empty plasmid, pEF1α-Myc, emitted no fluorescence.

### Distribution of IFI6 in different chicken tissues

**FIGURE 5:**
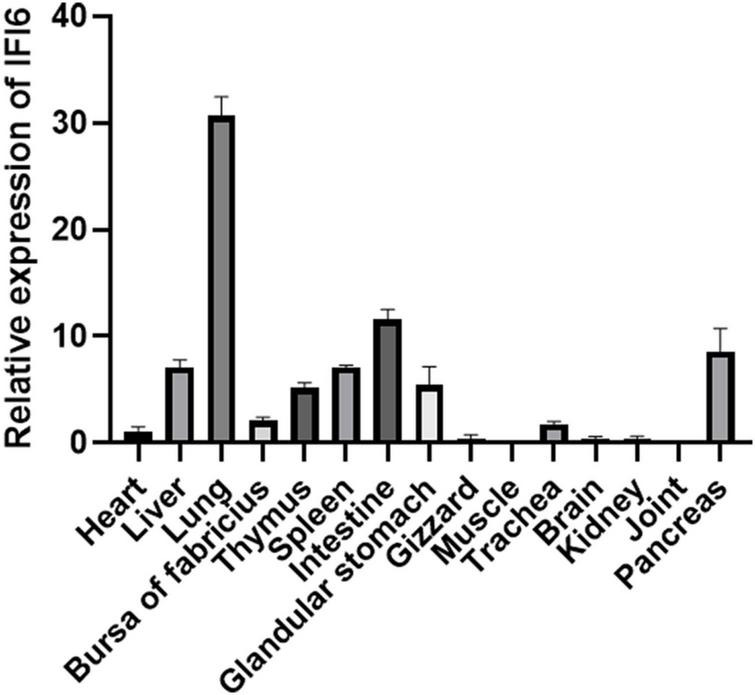
Expression analysis of chicken IFI6 in various tissues. Real-time fluorescence quantitative PCR was used to measure the IFI6 mRNA levels in the heart, liver, lung, bursa of Fabricius, thymus, spleen, glandular stomach, gizzard, muscle, trachea, brain, kidney, joint and pancreas of 14-day-old specific-pathogen-free chickens.

Real-time fluorescence quantitative PCR was used to detect the distribution of IFI6 in different tissues of 14-day-old specific-pathogen-free chickens. The highest expression of IFI6 was found in the lung, followed by the intestine, pancreas, liver, spleen, glandular stomach, thymus, bursa of Fabricius and trachea; a weak signal was detected in the heart, kidney, brain, gizzard and joints; and the lowest signal was detected in muscle (Figure 5).

### High expression of IFI6 reduced ARV replication

**FIGURE 6:**
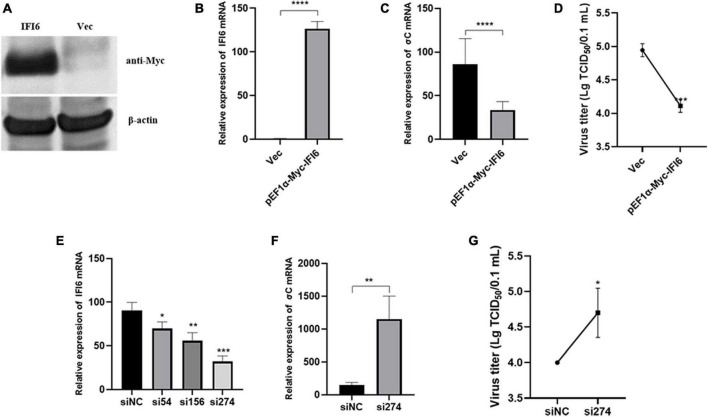
Interferon-alpha inducible protein 6 (IFI6) impedes ARV replication in DF-1 cells. DF-1 cells were transfected with pEF1α-Myc-IFI6 or pEF1α-Myc (Vec). High IFI6 expression in DF-1 cells was confirmed by Western blotting **(A)** and real-time fluorescence quantitative PCR **(B)**. DF-1 cells were transfected with pEF1α-Myc-IFI6 or pEF1α-Myc (Vec) and then infected with ARV at a multiplicity of infection (MOI) of 1. The number of virions was assessed by real-time fluorescence quantitative PCR **(C)**, and the viral titer **(D)** was measured 24 h after infection. DF-1 cells were transfected with IFI6-targeting short interfering RNA (siRNA) and untargeted control siRNA (siNC), and the interference efficiencies of the three siRNAs were determined by real-time fluorescence quantitative PCR and compared **(E)**. DF-1 cells were transfected with si274 or siNC and then infected with ARV at an MOI of 1; the number of virions was assessed by real-time fluorescence quantitative PCR **(F)**, and the virus titer **(G)** was measured 24 h after infection. The data are presented as the means ± standard deviations of three independent experiments. *P < 0.05, **P < 0.01, ***P < 0.001, and ****P < 0.0001.

To examine the effect of IFI6 on ARV replication, the pEF1α-Myc-IFI6 plasmid was transfected into DF-1 cells, and the cells were then infected with ARV. Twenty-four hours after infection, the expression of the target protein was verified by Western blotting and real-time fluorescence quantitative PCR. Due to the lack of an IFI6 monoclonal antibody, anti-Myc monoclonal antibody tags fused to the N-terminus of IFI6 were used to detect the expression of the target proteins. IFI6 protein expression (approximately 11 kDa) was confirmed in the cells transfected with pEF1α-Myc-IFI6, and target protein expression was not detected in the cells transfected with the empty vector (Vec) (Figure 6A). Real-time fluorescence quantitative PCR was used to measure the relative expression of IFI6 24 h after infection, and the expression level was found to be increased by approximately 196-fold in the experimental group compared with the control group (Figure 6B). As shown in Figure 6C, the viral load, which is represented by the σC gene level of ARV as measured by real-time fluorescence quantitative PCR, was significantly decreased after infection (*P* < 0.0001). In addition, the supernatant of the cells transfected with pEF1α-Myc-IFI6 showed a lower virus level than that of the control group cells, as determined by a viral titer assay (Figure 6D). Although viral proliferation was observed, the overexpression of IFI6 significantly inhibited ARV replication compared with that observed in control cells.

Therefore, we hypothesized that IFI6 negatively regulates ARV replication and that the inhibition of IFI6 expression promotes ARV replication. Three different siRNAs were used to downregulate IFI6 expression, and of these, si274 exhibited the greatest inhibitory effect (Figure 6E). Therefore, si274 was transfected into DF-1 cells, and the cells were then infected with ARV. Both the RNA and virus titers were significantly increased (*P* < 0.01 or *P* < 0.05), demonstrating that the downregulation of IFI6 expression increased ARV amplification in cells (Figures 6F, G). Taken together, these results suggest that ARV infection stimulates upregulation of the expression of IFI6 and that IFI6 exhibits anti-ARV activity.

### Effects of IFI6 on innate immune signaling pathway-related factors during ARV infection

**FIGURE 7:**
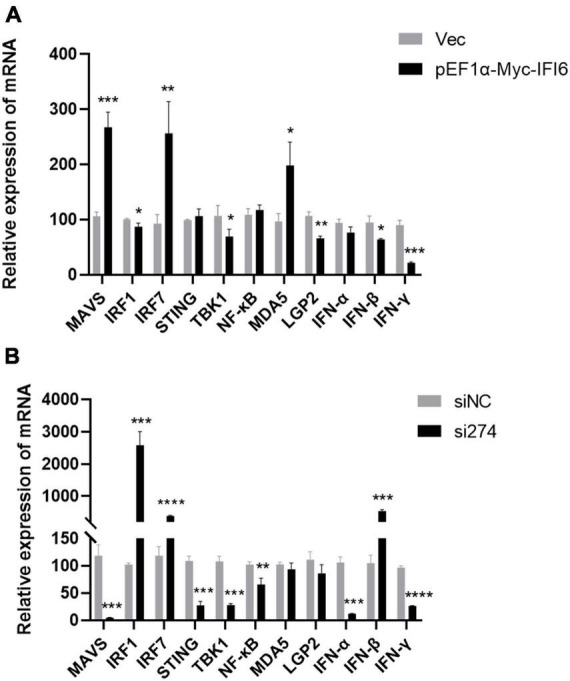
Effects of IFI6 on the expression of innate immune signaling pathway-related genes during ARV infection. DF-1 cells were transfected with pEF1α-Myc-IFI6 or pEF1α-Myc (Vec) and then infected with ARV at a multiplicity of infection (MOI) of 1. Twenty-four hours after infection, the mRNA expression levels of MAVS, IRF1, IRF7, STING, TBK1, NF-κB, MDA5, LGP2, IFN-α, IFN-β, and IFN-γ were measured by real-time fluorescence quantitative PCR **(A)**. DF-1 cells were transfected with short interfering RNA (si274) or untargeted control siRNA (siNC) and then infected with ARV at an MOI of 1. Twenty-four hours after infection, the MAVS, IRF1, IRF7, STING, TBK1, NF-κB, MDA5, LGP2, IFN-α, IFN-β, and IFN-γ mRNA levels were measured by real-time fluorescence quantitative PCR **(B)**. The results are presented as the means ± standard deviations of three independent experiments. *P < 0.05, **P < 0.01, ***P < 0.001, and ****P < 0.0001.

The aforementioned results suggest that chicken IFI6 inhibits ARV replication, but the relevant mechanism remains unknown. To explore the mechanism, we transfected the pEF1α-Myc-IFI6 recombinant plasmid and si274 against IFI6 into DF-1 cells, overexpressed or inhibited the expression of the IFI6 gene in these DF-1 cells, infected the cells with ARV 24 h after transfection, and collected infected cell samples 24 h after infection. The effect of IFI6 on the expression of innate immune signaling pathway-related factors after ARV infection was assessed by real-time fluorescence quantitative PCR, and the results are shown in Figure 7. IFI6 overexpression and knockdown significantly increased the IRF7 mRNA expression level (*P* < 0.01 or *P* < 0.0001) and downregulated the TBK1, LGP2, IFN-α, and IFN-γ mRNA expression levels (*P* < 0.05, *P* < 0.01, *P* < 0.001, or *P* < 0.0001). MDA5, MAVS, STING, and NF-κB mRNA expression was upregulated after IFI6 overexpression and downregulated after IFI6 knockdown (*P* < 0.05, *P* < 0.01, or *P* < 0.001). The expression levels of IRF1 and IFN-β mRNA were significantly downregulated after IFI6 overexpression and significantly upregulated after IFI6 knockdown (*P* < 0.05 or *P* < 0.001). Therefore, we hypothesized that changes in IFI6 expression may affect the expression of MDA5, MAVS, STING, NF-κB, IRF1, and IFN-β in ARV infection but may not affect IRF7, TBK1, LGP2, IFN-α, and IFN-γ expression or that IFI6 may either enhance or attenuate the ARV-mediated regulation of IRF7, TBK1, LGP2, IFN-α, and IFN-γ expression.

## Discussion

Interferon-alpha inducible protein 6 is an ISG. In this study, we cloned the IFI6 gene and compared the full CDS of the cloned IFI6 gene with the corresponding nucleotide sequences of corresponding genes in other species. This analysis revealed that the cloned sequence was completely consistent with the corresponding sequence in *G. gallus*. The IFI6 nucleotide sequence showed 73.6 and 87.4% homology with the corresponding sequences in *M. nubicus* and *T. guttata*, respectively, and its homology with the corresponding amino acid sequences in *H. sapiens*, *B. taurus*, *O. aries*, *S. scrofa* and other mammals did not exceed 50%. Multiple alignment analysis of the deduced amino acid sequence indicated 67.6 and 83.1% similarity with the *M. nubicus* and *T. guttata* sequences, respectively, and its similarity with *H. sapiens*, *B. taurus*, *O. aries*, *S. scrofa* and other mammals did not exceed 30%. These findings were clearly reflected in the evolutionary tree. Mammals and birds were clearly divided into two branches. Chickens and other birds showed more similar evolutionary histories and greater homology. This finding is consistent with the recognized evolutionary history and reflects the high variability of the IFI6 gene, suggesting that the IFI6 gene may have undergone strong natural selection during evolution. In addition, the chicken IFI6 gene is located in a different branch than the mammalian IFI6 gene, suggesting that its function in birds during viral infection may be different from that in mammals. The protein structure of IFI6 was predicted to be similar to that of IFI27L1, with a coverage rate of 51%. IFI27L1 also known as FAM14B or ISG12c, it consists of 104 amino acids with a molecular weight of about 9.5 kDa and can encode a small hydrophobic protein. IFI27L1 belongs to the FAM14 family along with IFIT6 (Parker and Porter, 2004; Cheriyath et al., 2011). The sequence that is similar between IFI6 and IFI27L1 is the transmembrane region. Understanding the related functions of IFI27L1 is of guiding significance for the study of IFI6. A few literatures have shown that IFI27L1 is a protein that can promote apoptosis (Xie et al., 2022). This provides clues for future research on the function of avian IFI6. The function of a protein is related not only to its structure but also to its distribution upon expression (Yang et al., 2015). Recent studies have found that the human IFI6 protein is located in the endoplasmic reticulum in human hepatoma cells and blocks yellow fever virus replication by blocking the invagination of the endoplasmic reticulum membrane (Richardson et al., 2018). The porcine IFI6 protein is located in the membrane of muscle cells, and its mRNA is abundantly expressed in muscle, where it is expressed at a significantly higher level than it is in the brain. IFI6 is a candidate gene for use to increase the pork quality (Kayan et al., 2011). The shrimp IFI6 protein is localized to the cytoplasm of Drosophila embryonic cells, and its mRNA expression is highest in the intestine, followed by the hepatopancreas, stomach, heart and gills, whereas low expression is observed in the epithelium, pyloric cecum, blood cells, nerves, muscles and eye stalks, which indicates that this protein plays a key role in defense against white spot syndrome virus in shrimp (Lu et al., 2022). We also studied the distribution of the expression of chicken IFI6. IFI6 was transfected into DF-1 cells and found to be a cytoplasmic protein. IFI6 mRNA expression was highest in the chicken lung, followed by the intestine, pancreas, liver, spleen, glandular stomach, thymus, bursa of Fabricius and trachea; weak signals were detected in the heart, kidney, brain, gizzard and joints; and the lowest expression was observed in the chicken muscle. These findings indicate that the IFI6 gene exhibits obvious tissue and species specificity, leading to the hypothesis that its functions also differ among different species.

Avian reovirus is mainly transmitted horizontally through the fecal-oral route but also vertically through eggs. After infection, ARV can replicate in the thymus, liver, spleen, bursa of Fabricius, intestine and other tissues and organs and can cause many diseases, such as hepatitis, enteritis, arthritis, myocarditis, and atrophy of the thymus and bursa of Fabricius (Roessler and Rosenberger, 1989; Wang et al., 2022). The distribution of innate immune-related genes in different parts of the host body is closely related to its antiviral effect (Cheng et al., 2015; Lin et al., 2021). In this study, we examined the expression of IFI6 in various tissues and organs of specific-pathogen-free chickens. We found that IFI6 was highly expressed in immune-related organs (spleen, thymus, and bursa of Fabricius), respiratory tract-related organs (lung and trachea), and digestive tract-related organs (pancreas, liver, intestine, and glandular stomach). Therefore, we speculate that IFI6 may play an important role in the innate immune response against ARV infection in chickens.

In addition, the overexpression of IFI6 in human liver cancer cells promotes hepatitis C virus replication and weakens the antiviral activity of IFN-α (Chen et al., 2016), and the same findings have been observed in liver cancer cells. IFI6 inhibits hepatitis B virus replication by binding to the promoter of hepatitis B virus (Sajid et al., 2021). IFI6 plays different roles in different viral infections, and it has been speculated that the antiviral effect of IFI6 is specific.

To study the antiviral effect of avian IFI6, we overexpressed and inhibited IFI6 expression in DF-1 cells and then infected these cells with ARV. IFI6 overexpression inhibited viral replication, and IFI6 inhibition promoted viral replication (as determined by the σC expression level), indicating that avian IFI6 exhibits an antiviral function. To further understand the antiviral role of IFI6, we assessed the expression levels of innate immunity-related molecules and found that the overexpression and inhibition of IFI6 upregulated and downregulated the expression levels of various innate immunity-related molecules, respectively.

The host’s pattern recognition receptors play an important role in sensing pathogen invasion. The current study found that the reovirus family is recognized by RIG-I or MDA5 (Kato et al., 2008). Only MDA5 and LGP2 are found in poultry, and RIG-I is missing (Kato et al., 2008; Barber et al., 2010); thus, in this study, we examined two nucleic acid receptors, MDA5 and LGP2. The results showed that the expression of MDA5 was upregulated after IFI6 overexpression and downregulated after IFI6 inhibition, and the expression of LGP2 was always downregulated. It is speculated that IFI6 may affect induction of the expression of MDA5 and MAVS.

In our study, overexpression or inhibition of IFI6 upregulated IRF7 expression and downregulated TBK1, LGP2, IFN-α, and IFN-γ expression after infection with ARV. The upregulated or downregulated expression of these factors was identified in comparison with the corresponding controls, which were different, and thus, the magnitude of the changes after overexpression or inhibition could not be compared to determine the involvement of IFI6 in the regulation of IRF7, TBK1, LGP2, IFN-α, and IFN-γ expression after infection with ARV. We hypothesize that changes in IFI6 expression may not affect the expression of IRF7, TBK1, LGP2, IFN-α, and IFN-γ in ARV infection and may also enhance or weaken the regulation of IRF7 and IFN-γ expression by ARV.

We were more interested in the changes in the expression of MAVS, IRF1 and IFN-β. The differences in the MAVS, IRF1, and IFN-β levels were notable. After IFI6 was overexpressed in cells that were then infected with ARV, MAVS expression was significantly upregulated, and IRF1 and IFN-β expression was significantly downregulated. After IFI6 knockdown and ARV infection, MAVS expression was significantly downregulated, and the expression of IRF1 and IFN-β was significantly upregulated.

As an important linker molecule in the innate immune signal transduction pathway that mainly recognizes RNA viruses, MAVS plays a crucial role in the innate immune response (Ren et al., 2020). For example, after RIG-I-like receptors recognize the virus, they interact with MAVS, and this interaction triggers a series of signal transduction cascades and ultimately leads to the expression of various proinflammatory factors and antiviral genes, such as IFNs and ISGs, which inhibit viral replication and spread (Meylan et al., 2005; Seth et al., 2005; Xu et al., 2005; Kumar et al., 2006; Song et al., 2019). MAVS exhibits an expression pattern similar to that of IFI6; presumably, ARV infection significantly upregulates the expression of IFI6, and after upregulation, IFI6 regulates the expression of MAVS and thus affects antiviral signaling cascades.

IRF1 is the first member of the IRF family found to activate type I IFN genes via their promoters (Dou et al., 2014). Studies have shown that IRF1 drives reprogramming of bone marrow dendritic cells and macrophages and thus triggers an antiviral signaling pathway; that is, IRF1 interacts with myeloid differentiation factor 88 or IL-1 receptor-related kinase-1 to activate IFN-β (Schmitz et al., 2007). In this study, IRF1 and IFN-β showed similar expression patterns, and in contrast to IFI6, ISGs negatively regulate the immune response of IFNs, such as SOCS1 and USP18, two well-characterized ISGs that negatively regulate IFN signaling by inhibiting the JAK-STAT signaling pathway (Malakhov et al., 2002; Malakhova et al., 2006; Yoshimura et al., 2007; William et al., 2014; Yu et al., 2018). Therefore, we hypothesized that IFI6 negatively regulates the JAK-STAT signaling pathway by inhibiting the expression of IFN-β. These findings provide clues for improving our understanding of the function of IFI6, revealing the signaling pathways involving IFI6, and laying a theoretical foundation for understanding the pathogenesis of ARV infection and for blocking ARV replication. To acquire a greater understanding of IFI6 function, further investigation is needed.

## Data availability statement

The original contributions presented in the study are included in the article/supplementary material, further inquiries can be directed to the corresponding author.

## Ethics statement

The animal study was approved by the Animal Protection and Experiment Ethics Committee of Guangxi Veterinary Research Institute. The study was conducted in accordance with the local legislation and institutional requirements.

## Author contributions

ZXX designed and co-ordinated the study and helped review the manuscript. LW performed the experiments, analyzed the data, and wrote the manuscript. SW assisted in completing the experiments and revised the manuscript. HR, LX, SL, ML, ZQX, QF, TZ, YZ, MZ, JH, and YW assisted with the animal experiments. All authors contributed to the article and approved the submitted version.

## Footnotes

1
https://npsa-prabi.ibcp.fr/cgi-bin/npsa_automat.pl?page=npsa_gor4.html

2
https://swissmodel.expasy.org/interactive
